# Prognostic Factors Toward Clinically Relevant Radiographic Progression in Patients With Rheumatoid Arthritis in Clinical Practice: A Japanese Multicenter, Prospective Longitudinal Cohort Study for Achieving a Treat-to-Target Strategy

**DOI:** 10.1097/MD.0000000000003476

**Published:** 2016-04-29

**Authors:** Tomohiro Koga, Akitomo Okada, Takaaki Fukuda, Toshihiko Hidaka, Tomonori Ishii, Yukitaka Ueki, Takao Kodera, Munetoshi Nakashima, Yuichi Takahashi, Seiyo Honda, Yoshiro Horai, Ryu Watanabe, Hiroshi Okuno, Toshiyuki Aramaki, Tomomasa Izumiyama, Osamu Takai, Taiichiro Miyashita, Shuntaro Sato, Shin-ya Kawashiri, Naoki Iwamoto, Kunihiro Ichinose, Mami Tamai, Tomoki Origuchi, Hideki Nakamura, Kiyoshi Aoyagi, Katsumi Eguchi, Atsushi Kawakami, the Japanese RA Patients with RRP Study Group

**Affiliations:** From the Unit of Translational Medicine, Department of Immunology and Rheumatology, Nagasaki University Graduate School of Biomedical Sciences (TK,YH, S-YK, NI, KI, MT, HN, AK); Japanese Red Cross Nagasaki Genbaku Hospital, Department of Rheumatology, Nagasaki (AO, MN, TA); Department of Rheumatology, Kurume University Medical Center, Kurume (TF, MN); Zenjinkai Shimin-no-Mori Hospital, Miyazaki (TH); Department of Hematology and Rheumatology, Tohoku University Hospital, Sendai (TI, RW); Rheumatic and Collagen Disease Center, Sasebo Chuo Hospital, Sasebo (YU); Tohoku Pharmaceutical University Hospital, Sendai (TK); Yu Family Clinic, Miyagi (YT); Kurume University School of Medicine, Kurume (SH); Department of Orthopaedic Surgery, Tohoku University Hospital (HO); East Sendai Rheumatism and Internal Medicine Clinic, Sendai (TI); Osaki Citizen Hospital, Osaki (OT); NHO Nagasaki Medical Center, Omura (TM); Clinical Research Center, Nagasaki University Hospital (SS); Department of Public Health (S-YK, KA); Department of Rehabilitation Sciences, Nagasaki University Graduate School of Biomedical Sciences, Nagasaki (TO); and Sasebo City General Hospital, Sasebo (KE), Japan.

## Abstract

To determine prognostic factors of clinically relevant radiographic progression (CRRP) in patients with rheumatoid arthritis (RA) in clinical practice.

We performed a multicenter prospective study in Japan of biological disease-modifying antirheumatic drug (bDMARD)-naive RA patients with moderate to high disease activity treated with conventional synthetic DMARDs (csDMARDs) at study entry. We longitudinally observed 408 patients for 1 year and assessed disease activity every 3 months. CRRP was defined as yearly progression of modified total Sharp score (mTSS) > 3.0 U. We also divided the cohort into 2 groups based on disease duration (<3 vs ≥3 years) and performed a subgroup analysis.

CRRP was found in 10.3% of the patients. A multiple logistic regression analysis revealed that the independent variables to predict the development of CRRP were: CRP at baseline (0.30 mg/dL increase, 95% confidence interval [CI] 1.01–1.11), time-integrated Disease Activity Score in 28 joints-erythrocyte sedimentation rate (DAS28-ESR) during the 1 year postbaseline (12.4-unit increase, 95%CI 1.17–2.59), RA typical erosion at baseline (95%CI 1.56–21.1), and the introduction of bDMARDs (95%CI 0.06–0.38). The subgroup analysis revealed that time-integrated DAS28-ESR is not a predictor whereas the introduction of bDMARDs is a significant protective factor for CRRP in RA patients with disease duration <3 years.

We identified factors that could be used to predict the development of CRRP in RA patients treated with DMARDs. These variables appear to be different based on the RA patients’ disease durations.

## Abstract 2

Supplemental Digital Content is available in the text

## INTRODUCTION

Rheumatoid arthritis (RA) is a chronic inflammatory disease characterized by autoimmune disorder and the destruction of synovial joints, leading to impaired quality of life and premature mortality.^1,2^ The current therapeutic strategy for RA has developed remarkably, and the 2010 European League Against Rheumatism (EULAR) recommendations for the management of RA were updated in 2013.^3^ These recommendations describe a treat-to-target (T2T) approach using conventional synthetic disease-modifying antirheumatic drugs (csDMARDs) in phase 1 followed by the addition of a biological disease-modifying antirheumatic drug (bDMARD) or another csDMARD in phase 2.

Diagnostic techniques for the management of RA have also advanced. Magnetic resonance imaging (MRI) and ultrasonography (US) are sensitive enough to detect active synovitis and erosions in early RA.^4–7^ Nevertheless, conventional plain radiography of the hands and feet is still considered the gold standard for the assessment of joint damage progression and the efficacy of treatment.^8,9^ In particular, modified Sharp/van der Heijde analyses have been used in the majority of clinical trials.^10–12^

The primary goal of RA treatment is to control disease activity and prevent structural damage, but some patients develop clinically relevant radiographic progression (CRRP) despite conventional treatment with DMARDs. In these patients, a treatment strategy providing strict control of the progression of RA should be considered in order to alter the course of radiographic progression.^13,14^ Accordingly, the identification of individual RA patients at high risk of CRRP is critical for achieving the goal of RA control. Various clinical and biological markers including C-reactive protein (CRP), the erythrocyte sedimentation rate (ESR), and the presence of autoantibodies have been identified as risk factors for CRRP in patients with RA, especially those enrolled in clinical trials treated with bDMARDs.^15–18^

These cohort studies adopted tender joint counts and swollen joint counts as clinical indices. The matrix models based on these variables did not include a commonly used composite measure such as the Disease Activity Score in 28 joints (DAS28). Some of the studies of RA cohorts in clinical practice have investigated a CRRP model,^19,20^ but to the best of our knowledge there has been no large-scale clinical study investigating the prevention of CRRP by using RA patients treated in accord with the EULAR recommendations in daily clinical practice.

To assess the relevance of the updated EULAR recommendations and to determine prognostic factors of CRRP in Japanese RA patients in clinical practice, we conducted a large-scale prospective study and evaluated the associations between clinical variables and the risk of developing CRRP among csDMARD-refractory RA patients.

## METHODS

### Study Population

This was a prospective, observational cohort study registered with the University Hospital Medical Information Network Clinical Trials Registry [http://www.umin.ac.jp/ctr/] (#UMIN000014791), conducted in the daily clinical practice for RA in Japan. The inclusion criteria were as follows: RA patients who met the American College of Rheumatology (ACR) 1987 criteria or the 2010 RA classification criteria;^1,21^ the patient's clinical disease activity determined by the DAS28-ESR is moderate to high or, obvious plain radiographic erosion is confirmed at enrollment; and RA patients taking csDMARDs but not bDMARDs at enrollment.

Overall, 887 patients with csDMARDs-refractory RA from 26 related centers of Nagasaki University and Tohoku University in Japan were recruited in our cohort between May 2009 and March 2012. All of the patients were examined and treated by Japan College of Rheumatology-certified rheumatologists. Although this was a prospective, observational cohort study, we recommended that all of the participating rheumatologists treat the patients using a T2T strategy. We did not recommend the choice of DMARDs. We observed the patients for 1 year after enrollment and assessed the RA disease activity every 3 months, including use of the DAS28-ESR and the Health Assessment Questionnaire.^22^

According to the T2T strategy, physicians were allowed to introduce bDMARDs including infliximab, adalimumab (ADA), golimumab, certolizumab pegol, etanercept, tocilizumab (TCZ), and abatacepet (ABT) if the patient's disease activity was not controlled by csDMARDs alone. All of the above bDMARDs were available in Japan during the observational period. To determine the efficacy of bDMARDs for the prevention of CRRP in the present investigation, we selected the patients for whom a bDMARD was introduced within 3 months after their study enrollment. All patients gave their signed informed consent to be subjected to the protocol, which was approved by the Institutional Review Board of Nagasaki University, Tohoku University, and the related centers.

### Structural Damage Assessment

Radiographs of each patient's hands and feet were taken at baseline and at 1 year, and we evaluated the radiographic progression by determining the changes over the year by obtaining each patient's modified total Sharp score (mTSS), joint erosion, and joint space narrowing at 1 year.^23,24^ The images were scored by 2 independent rheumatologists, trained and certified by Prof van der Heijde (Leiden University Medical Center), and blinded to the clinical evaluation as described.^25,26^ The interobserver reliability (as determined by the interclass correlation coefficient) was 0.97. The smallest detectable change of mTSS in the present study was calculated as 2.96 as described.^27^ We therefore defined an annual increase of the mTSS > 3.0 units as the development of CRRP, according to a previous report.^28^ In addition, we considered the patients as having typical RA erosion if erosion score is ≥3 according to the EULAR definition of erosive disease.^29^

### Statistical Analysis

Baseline demographic, clinical, and radiographic characteristics of RA patients with or without CRRP were compared with Fisher exact tests for discrete variables and Wilcoxon test for continuous variables. Previous studies have shown that the titer of autoantibodies such as anticitrullinated peptide antibodies (ACPA) or rheumatoid factor (RF), especially the latter, is influenced by DMARDs.^30,31^ Since all of the patients at entry had already been treated by csDMARDs in the present study, the titer of autoantibodies may not represent the exact serologic characteristics. Therefore, we adopted the presence of ACPA/RF, instead of the titer, in the present study. To determine the independent predictive factors toward the development of CRRP at 1 year, we subsequently performed multiple logistic regression analysis. We selected variables with *P*-values <0.3 by univariate analyses as model 1. We then set model 2 by including variables with *P*-values <0.05 in model 1.

To test the interaction variables, we created model 3 by adding interaction terms between treatment (early introduction of bDMARDs) and baseline disease parameters (CRP at baseline and disease duration) as independent variables. We finally determined model 2 as the final model.

We also performed subgroup analysis based on disease duration (<3 vs ≥3 years). Each subgroup was analyzed with a logistic regression analysis by using the final model. In order to adjust for nonrandom assignment to bDMARDs treatment, we further analyzed using propensity scores. Variables used in constructing the propensity score were age, gender, disease duration, CRP at baseline, erosion score ≥3 at baseline, methotrexate (MTX) use at baseline, and Prednisolone use. Statistical analyses were performed using SAS 9.4 software (SAS Institute, Cary, NC). A *P*-value <0.05 was considered significant.

## RESULTS

### Patients

**FIGURE 1:**
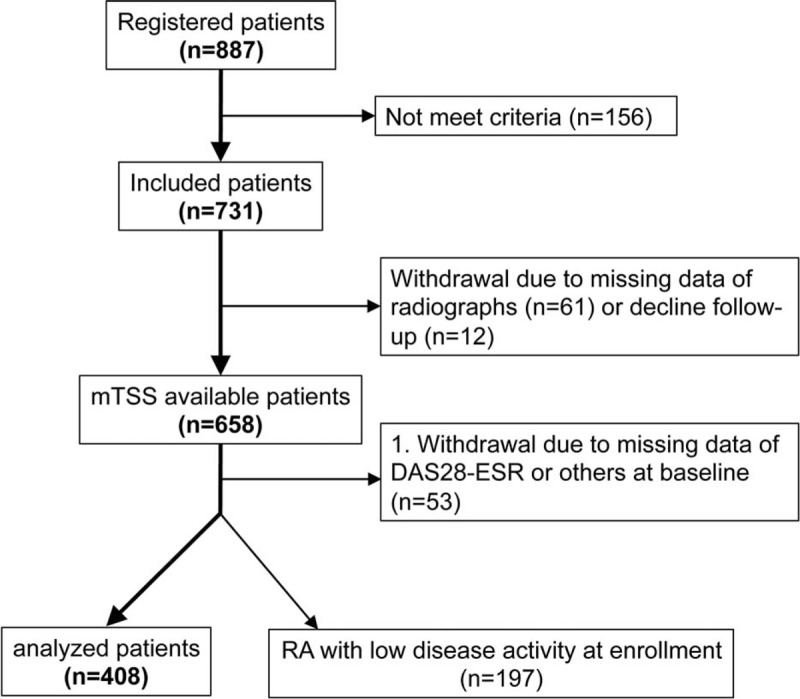
Patient enrollment flow chart. DAS28-ESR = Disease Activity Score in 28 joints-erythrocyte sedimentation rate, mTSS = modified total Sharp score.

Figure 1 is the flow chart of the patient enrollment: 731 of the 887 registered patients met the inclusion criteria and were enrolled in our cohort. Since we performed a complete case analysis, cases with any missing data at baseline were excluded in our study. Clinical demographics in between enrolled case (n = 605) and excluded cases (n = 53) were not significantly different (data not shown). As a result, 605 had complete available data at 1 year, and we finally analyzed 408 patients with moderate to high disease activity at enrollment in this study. The patients’ characteristics are shown in Table 1.

**TABLE 1:**
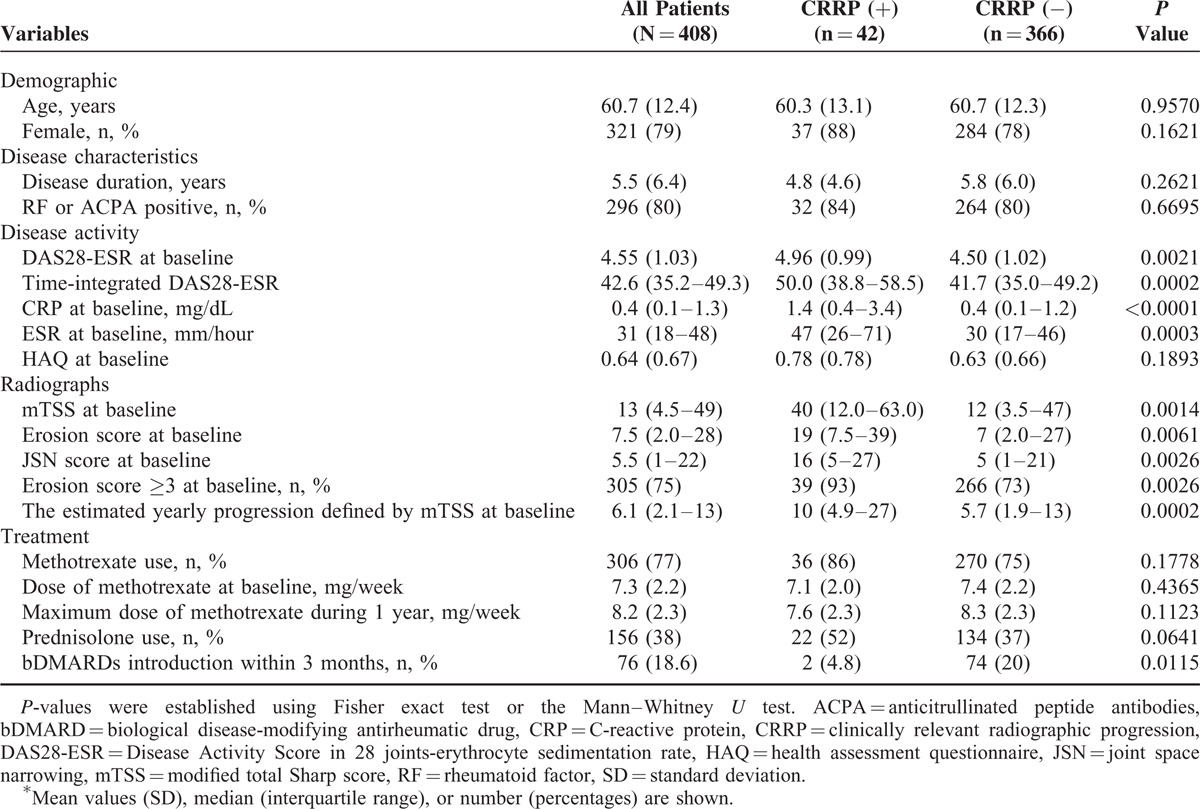
Association Between Baseline Characteristics and CRRP (Univariate Analyses)^∗^

The patients’ mean age was 60.7 years, and the mean disease duration was 5.5 years. During the 1-year observation period, the treatment of 249 patients was strengthened with 1 or more csDMARDs or bDMARDs according to the EULAR recommendation, as decided by the participating rheumatologists. Among these 249 patients, the dosage of csDMARDs for 124 patients was increased. For 43 patients, the csDMARD(s) were switched or 1 or more other csDMARD was added. For 85 patients, a bDMARD was initiated after study entry. The average period before the introduction of a bDMARD was 2.5 months. bDMARDs (ADA, n = 23; ETA, n = 17; infliximab, n = 14; TCZ, n = 10; ABT, n = 7; and golimumab, n = 5) were initiated within 3 months after the enrollment for 76 patients. During the study, 12 patients dropped out; the retention rate of csDMARDs or bDMARDs was >90%. The therapeutic course during the 1 year following the baseline is shown in Supplementary Figure S1.

### Radiographic Progression

**FIGURE 2:**
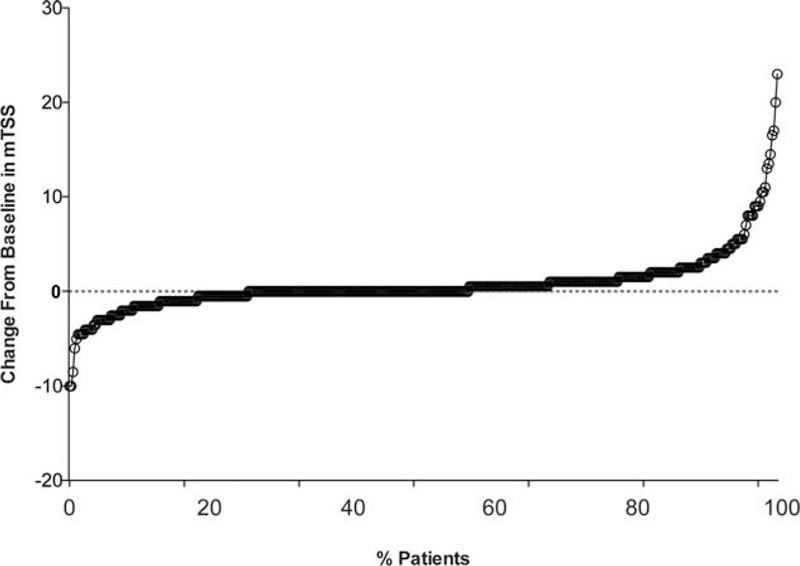
Cumulative probability plots of actual radiographic progression assessed by modified total Sharp score (mTSS, U/year) in the cohort (n = 408).

CRRP was observed in 42 of the 408 patients (10.3%). Cumulative probability plots during the 1 year postbaseline as assessed by mTSS are shown in Figure 2.

### Prediction of CRRP at 1 Year in 408 RA Patients

To determine which variables are associated with the development of CRRP at 1 year, we evaluated 14 variables as shown in Table 1. We found that the 7 variables significantly associated with CRRP in the univariate analyses were DAS28-ESR at baseline, time-integrated DAS28-ESR during the 1 year postbaseline, CRP at baseline, ESR at baseline, total mTSS at baseline, erosion score ≥3 at baseline, and the introduction of bDMARDs within 3 months of enrollment. We subsequently selected the independent variables with *P*-values <0.3 by univariate analyses and a performed logistic regression analysis, which revealed 5 independent variables that could be used to predict the development of CRRP, as follows: disease duration, time-integrated DAS28-ESR during the 1 year postbaseline, CRP at baseline, erosion score ≥3 at baseline, and the introduction of bDMARDs.

**FIGURE 3:**
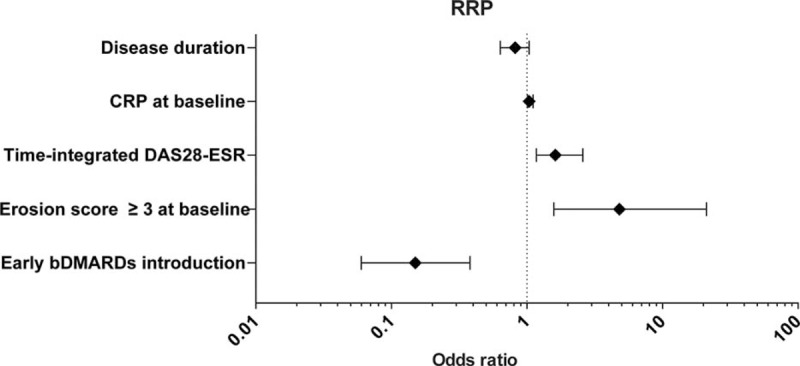
Prediction of CRRP at 1 year postbaseline in 408 RA patients by the logistic regression analysis. bDMARD = biological disease-modifying antirheumatic drug, CRP = C-reactive protein, CRRP = clinically relevant radiographic progression, DAS28-ESR = Disease Activity Score in 28 joints-erythrocyte sedimentation rate, RA = rheumatoid arthritis.

To this end, we determined the final model by selecting the variables with *P*-values <0.05 in the 1st model and found 4 prognostic factors of CRRP, as follows: CRP at baseline (0.30 mg/dL increase, odds ratio [OR] = 1.04, 95% confidence interval [CI] 1.01–1.11, *P* = 0.01411), time-integrated DAS28-ESR during the 1 year postbaseline (12.4-unit increase, OR = 1.62, 95%CI 1.17–2.59, *P* = 0.00267), RA typical erosion (erosion score ≥3) at baseline (OR = 4.81, 95%CI 1.58–21.1, *P* = 0.01409), and the introduction of bDMARDs (OR = 0.15, 95%CI 0.06–0.38, *P* = 0.01477) (Figure 3).

Since the mean disease duration was significantly shorter and the mean serum CRP level at baseline was significantly higher in the early bDMARDs introduction group than in the other patients (the disease duration 5.8 vs 4.2 years, *P* = 0.0426; CRP 2.02 vs 1.09 mg/dL, *P* = 0.0110, respectively), we considered that these variables had their interaction. Accordingly, we included the interaction terms (disease duration × the introduction of bDMARDs and CRP at baseline × the introduction of bDMARDs) with the final model in a logistic analysis and found there is no significant interaction between these variables (disease duration × the introduction of bDMARDs, OR = 0.84, 95%CI 0.38–1.31, *P* = 0.49597; CRP at baseline × the introduction of bDMARDs, OR = 1.15, 95%CI 0.89–1.42, *P* = 0.16818).

In order to confirm the efficacy of bDMARDs treatment in this cohort, we further analyzed using propensity scores. We still found a significant effect of bDMARDs toward CRRP after adjusting propensity scores matching (data not shown).

### Prediction of CRRP at 1 Year Postbaseline in Subgroups Defined by Disease Duration

In our cohort, we found that the patients’ disease duration at baseline was associated with CRRP. There have been some reports showing the rate of radiographic progression is more rapid in the first 2 to 3 years of RA.^32,33^ We therefore speculated that the variables that could be used to predict CRRP might be different between early-stage RA and established RA. Accordingly, we divided the cohort into 2 groups based on disease duration (<3 vs ≥3 years; given the idea as described^34^) and conducted a subgroup analysis. The patients’ characteristics according to disease duration are shown in Table 2.

**TABLE 2:**
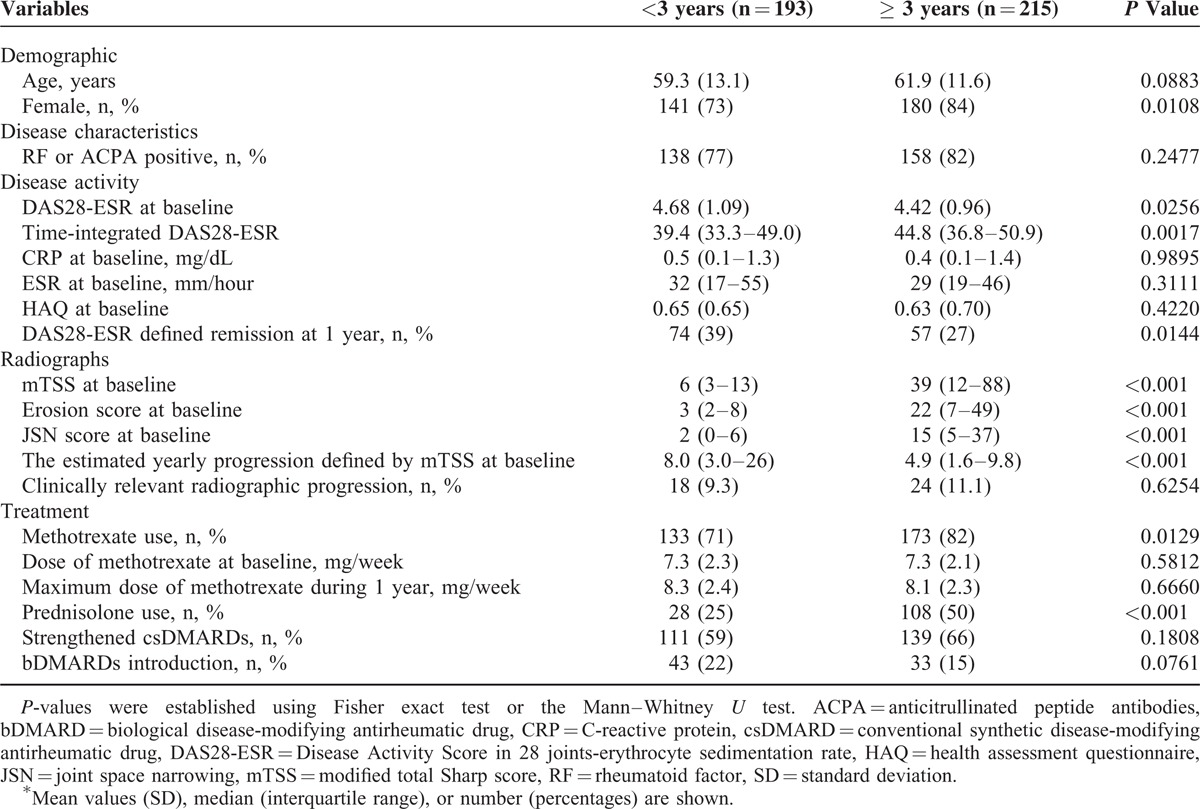
Patients’ Characteristics With Disease Duration (Univariate Analyses)^∗^

Although the DAS28-ESR values at baseline were significantly higher in the subgroup with disease duration <3 years (n = 193, median 0.84 years, interquartile range 0.29–1.85) compared to the ≥3 years group (n = 215, median 7.75 years, interquartile range 4.98–11.9) (mean DAS28-ESR; 4.68 vs 4.42, *P* = 0.0256), the rate of remission at 1 year was also significantly higher in the former group (39% vs 27%, *P* = 0.0144). The estimated yearly progression calculated by dividing mTSS at baseline by disease duration at baseline was significantly higher in the <3 years group compared to the ≥3years group (mean yearly progression; 8.0 vs 4.9, *P* < 0.001), whereas no significant difference was observed in the percentage of CRRP between the 2 groups (<3 years, 9.3%; ≥3 years, 11.1%; *P* = 0.6254).

**FIGURE 4:**
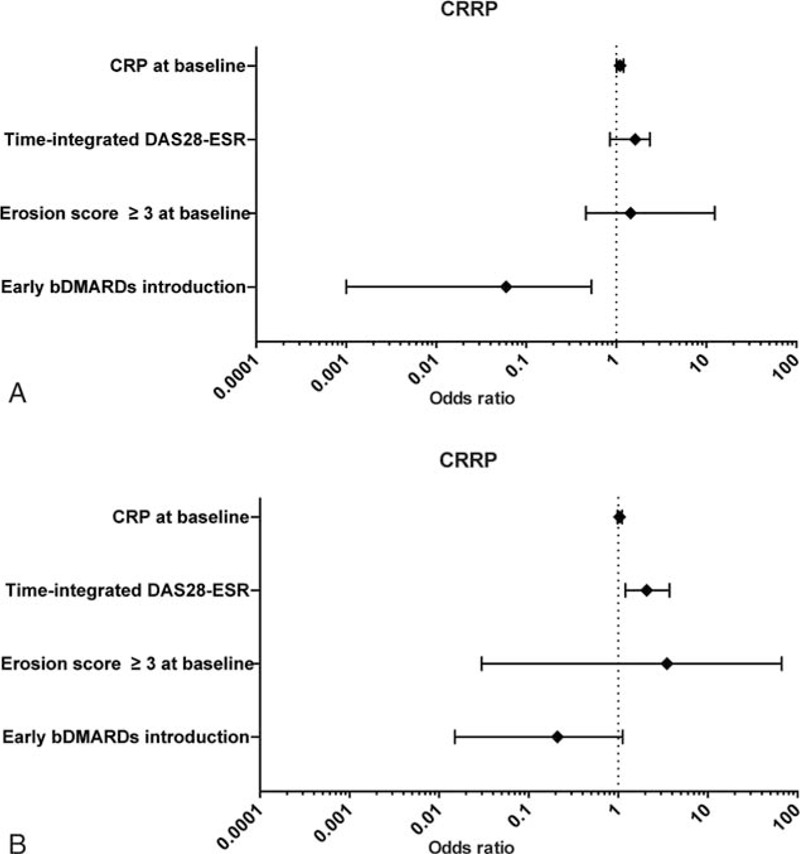
Prediction of CRRP at 1 year postbaseline in the subgroups defined by disease duration: <3 years (A, n = 193) or ≥3 years (B, n = 215) in the logistic regression analysis. bDMARDs = biological disease-modifying antirheumatic drug, CRP = C-reactive protein, CRRP = clinically relevant radiographic progression, DAS28-ESR = Disease Activity Score in 28 joints-erythrocyte sedimentation rate.

We performed a logistic regression analysis for each subgroup by using the final model created in this study. In the group with disease duration <3 years, CRP at baseline (0.30 mg/dL increase, OR = 1.10, 95%CI 1.01–1.20, *P* = 0.0306), and the introduction of bDMARDs (OR = 0.06, 95%CI 0.001–0.53, *P* = 0.0064) are independent variables to predict the development of CRRP (Figure 4A). In contrast, we found that time-integrated DAS28-ESR during the 1 year postbaseline (12.1-unit increase, OR = 2.05, 95%CI 1.20–3.62, *P* = 0.0081) was the only variable for predicting CRRP in the group with ≥3 years’ disease duration (Figure 4B). Taken together, our results show that the prognostic factors of CRRP differ according to the disease duration.

## DISCUSSION

Since structural damage is closely associated with functional disability in RA,^35,36^ CRRP has been one of the most important outcomes in both clinical trials and observational studies of RA patients.^12,25,26,37^ Our present study sought to assess the relevance of phase 1 and phase 2 treatment policy described in the EULAR recommendations and to determine the predictive factors for CRRP in Japanese patients with RA in daily clinical practice. In the entire cohort of 408 patients, we observed that the time-integrated DAS28-ESR values at 1 year postbaseline were associated with the development of CRRP, which confirms the importance of the T2T recommendations in the 2013 update of the EULAR recommendations.^3,38^

Our identification of the CRP level at baseline as the predictive factors for CRRP in the present study is consistent with previous studies.^17–20^ Our findings also provide evidence that the early introduction of bDMARDs significantly inhibits the development of CRRP. The 2013 update of the EULAR recommendations states that the tumor necrosis factor inhibitors, ABT and TCZ, show similar efficacy and safety,^3^ and thus we did not separate each class of bDMARDs. In the BeSt, ATTRACT, and ASPIRE clinical trials, the initial combinations of tumor necrosis factor inhibitors with MTX strongly inhibited the development of rapid radiographic progression compared to MTX monotherapy.^17,18^ However, in real-world practice, most bDMARDs are used to treat csDMARD-refractory patients, not csDMARD-naive patients. To our knowledge, the present study is the 1st longitudinal observational study that clearly shows the protective effect of the addition of bDMARDs against the development of CRRP in csDMARD-refractory RA patients. The DAS28-ESR values at baseline were clearly high even in our subgroups of bDMARDs users compared to the bDMARDs nonusers. This also reflects the remarkable protective effect of bDMARD against CRRP in RA.

It has been reported that ACPA or RF can be used as a predictive factor for CRRP,^17–20^ but these autoantibodies were not shown to be predictive in the present study. Possible reasons for this discrepancy are that: we included all of the consecutively treated RA patients, and thus the disease duration of the patients varied, whereas previous observations mostly targeted early-stage RA;^17–19^ we could not separate ACPA or RF and thus combined them to identify the autoantibodies-positive patients; and we were unable to examine the titers of ACPA or RF. Regarding to the titer of ACPA or RF toward radiographic progression in patients with RA, the opposite results have been published in the literature^17–20,39–41^ The disagreement might be induced by the difference or presence/absence of DMARDs treatments of the subjects at entry. However, we cannot exclude the possibility that the contributions of ACPA and/or RF were underestimated in the present study.

It seems quite important that the predictive factors for CRRP differ based on the RA disease duration. Since the clinical variables differed markedly by the disease duration cut-off of 3 years in the present study, we performed the subgroup analysis to clarify whether predictive factors for CRRP differ based on the disease duration, and we obtained notable insights. First, the effects of bDMARDs seem to be more potent at the early stage of RA.

In line with this observation, the DE019 clinical trial of ADA revealed that the efficacy of ADA toward ΔmTSS was greater among patients with RA of <3 years’ duration compared to those with ≥3 years’ duration.^34^ In the present study, the rate of DAS28-ESR remission at 1 year was significantly higher in the RA patients with <3 years’ disease duration compared to those with ≥3 years’ duration (<3 years, 39%; ≥3 years, 27%; *P* = 0.0144) whereas the rate of CRRP was comparable between these groups (<3 years, 9.3%; ≥3 years, 11.1%; *P* = 0.6254). In addition, time-integrated DAS28-ESR did not contribute to the development of CRRP in the RA patients with <3 years’ disease duration.

These data suggest that radiographic progression in RA (i.e., CRRP) may not be suppressed in early-stage RA patients only through the control of clinical disease activity, that is, DAS28-ESR. Thus, an evaluation by MRI or US is thought to be useful to predict the subgroup of patients who will develop CRRP among early-stage RA patients. The EULAR recommendations for the use of imaging of the joints in the clinical management of RA state, in recommendation #5, that MRI bone edema as well as the synovitis detected by MRI and US are strong independent predictors of subsequent radiographic progression in early RA and should be considered for use as a prognostic indicator.^7^ We consider that this statement is also applied in cases of CRRP.

In the present study, the estimated yearly progression of mTSS from baseline was significantly high in the RA subgroup with disease duration <3 years. These results are consistent with the well-known observation that radiographic progression occurs early (within the first 2–3 years) in patients with RA.^42^ Thus, taken together, the past and present findings indicate that physicians should pay close attention to the timing of bDMARD introduction – especially among early-stage RA patients – since bDMARDs can inhibit radiographic progression regardless of clinical disease activity.^43,44^

The time-integrated DAS28-ESR seems to be a more valuable predictive factor in established RA, since our multivariate analysis revealed that in our cohort, the time-integrated DAS28-ESR was not an independent predictive factor in the patients with <3 years’ disease duration but that it was an independent predictive factor in the patients with ≥3 years’ duration. We speculate that the speed of radiographic progression starts to decline in cases of established RA, and thus the control of clinical disease activities (i.e., the DAS28-ESR) is enough and essential in patients with ≥3 years’ RA duration. Nevertheless, long-term observation is needed to confirm these results, since a previous study showed that the time-integrated DAS28-ESR over 3 years is associated with CRRP in early RA patients.^45^

The dose of MTX in our Japanese cohort was lower than the other cohort. Previous clinical study has shown that the optimum dosage of MTX for a Japanese individual is 6 to 8 mg/week, and that higher dosages will increase the incidence of adverse effects.^46^ Furthermore, it has also been proposed that Japanese patients with RA showed high concentration of active form of MTX-polyglutamate in red blood cells with very low-dose MTX.^47^ Accordingly, it is assumed that patients in this cohort were treated according to the EULAR recommendations.

This study has some limitations. First, as stated above, a relatively short period of observation was used. The EULAR T2T recommendations state in recommendation #5 that the desired treatment target should be maintained throughout the remaining course of the disease.^38^ Thus, long-term verification studies are required to confirm the present results. Second, the mean DAS28-ESR at baseline in our study was 4.54, which seems to be low compared with the previous research providing the pilot risk model of rapid radiographic progression.^17–19^ Verification studies targeting RA patients with high clinical disease activity are thus also needed.

In conclusion, the present study is the first prospective observation to examine predictive variables of CRRP among Japanese RA patients being treated with DMARDs in daily practice. Our findings indicate that CRRP in our cohort was closely associated with the persistent disease activity, CRP at baseline, and the early introduction of bDMARDs. It is of note that some of these risk variables vary according to the disease duration. Our results provide useful information in relation to strict disease control by T2T strategies in accord with the EULAR recommendations 2013 update.

## Supplementary Material

Supplemental Digital Content
