# Supplementary material for: Prognostic Factors Toward Clinically Relevant Radiographic Progression in Patients With Rheumatoid Arthritis in Clinical Practice: A Japanese Multicenter, Prospective Longitudinal Cohort Study for Achieving a Treat-to-Target Strategy
Source: Medicine (Baltimore). 2016 Apr 29;95(17):e3476. doi: 10.1097/MD.0000000000003476 (PMC4998707; doi:10.1097/MD.0000000000003476)

Supplementary Fig. S1. The therapeutic course during the 1 year after the baseline in our RA cohort. bDMARDs = biological disease-modifying antirheumatic drug; csDMARDs = conventional synthetic disease-modifying antirheumatic drugs.


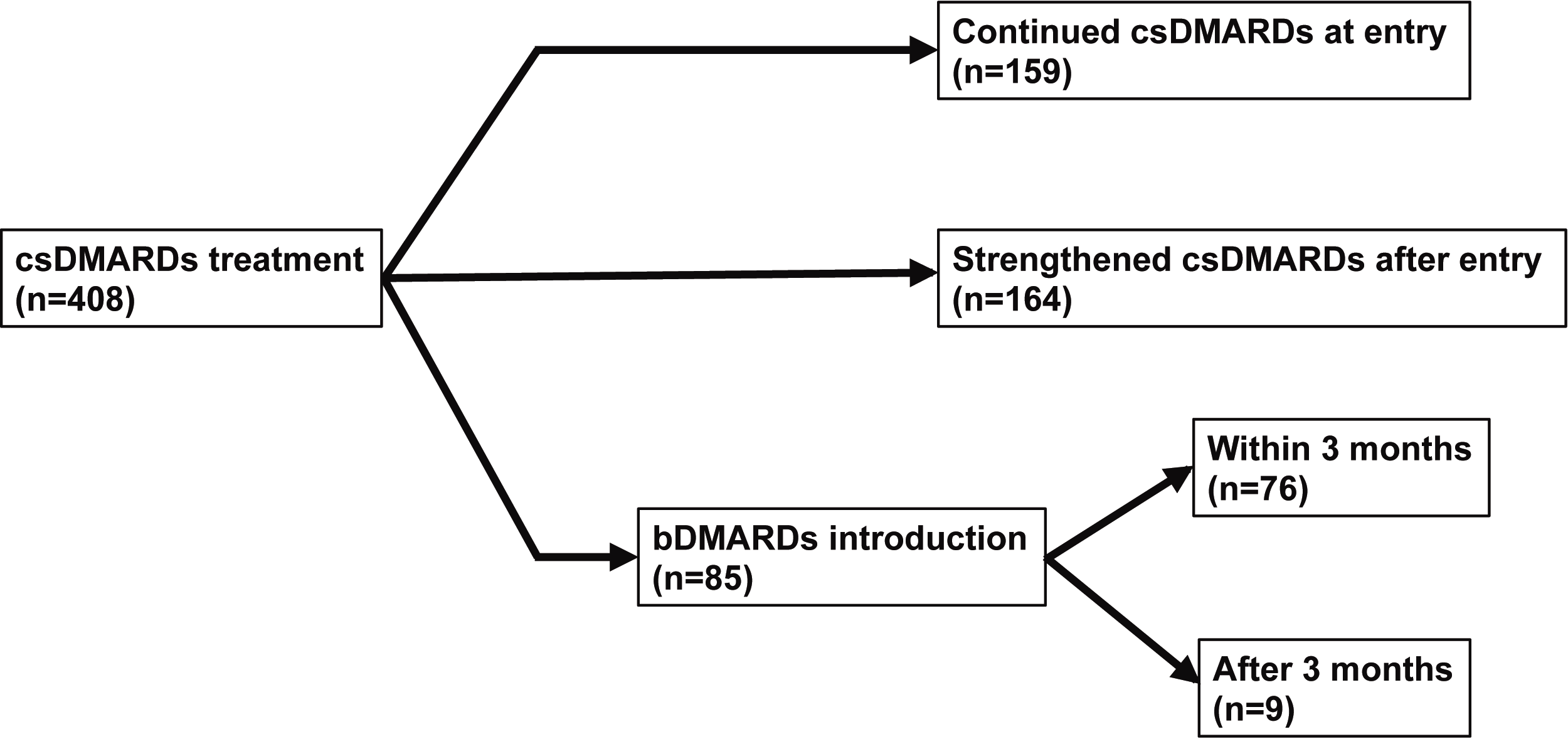

Supplement: Supplemental Digital Content [file medi-95-e3476-s001.doc]
